# Supporting the use of a coagulometric method for rivaroxaban control: a hypothesis-generating study to define the safety cut-offs

**DOI:** 10.1186/s12959-015-0058-9

**Published:** 2015-08-06

**Authors:** Raul Altman, Claudio Daniel Gonzalez

**Affiliations:** Centro de Trombosis de Buenos Aires, Buenos Aires, Argentina; Department of Pharmacology, School of Medicine, University of Buenos Aires, Buenos Aires, Argentina

**Keywords:** Anticoagulants, Drug monitoring, Reference ranges, Rivaroxaban, Safety

## Abstract

**Aims:**

Although quantitative anti-FXa assays can be used to measure rivaroxaban plasma levels, they are not widely performed or available. We aimed to tentatively determine the cut-off for thromboembolism and bleeding prevention based on the clotting effect of non-rivaroxaban conjugate-activated FX plasma levels in patients with rivaroxaban using a coagulometric method.

**Methods and results:**

Rivaroxaban was added *in vitro* to normal plasma at a range of 0 to 241 μg/L to cover expected peak and trough levels. Rivaroxaban chromogenic (μg/L) and RVV-confirm as a ratio were determined. Patient plasma samples were assayed with the RVV-confirm reagent. The appropriate rivaroxaban plasma concentration to inhibit clotting mechanisms was based on the remaining FXa in plasma, which was expressed as the ratio of patients/normal, R-C. There is a high correlation between R-C *in vitro and* spiked normal plasma rivaroxaban concentration (R-Square 0.910, linear equation; 0.971 quadratic equation, *p* < 0.0001 for both) but not with plasma rivaroxaban chromogenic assays. We propose a cut-off R-C value of 1.65 and 4.5 for safety. Based on the proposed therapeutic range, in 158 assays performed in 58 patients, 6.3 % assays were above the level of bleeding tendency at the peak (R-C 5.39 ± 1.01, median 5.13) and 42 % assays were below the prevention cut-off at the trough (R-C 1.31 ± 0.18, median 1.35).

**Conclusions:**

RVVconfirm® is fast and sensitive to measure the effect of rivaroxaban. Clinical studies are needed to establish whether this cut-off is useful for identifying patients at increased risk of hemorrhage or those who exhibit a low level of anticoagulation.

## Introduction

The well-established benefits of anticoagulant therapy are significantly hampered by the possibility of thromboembolism or major and sometimes fatal bleeding complications. These adverse effects can range from simple external skin bruising and bleeding (epistaxis, gastroduodenal bleeding, pulmonary complications) to problems in vital organs, including temporary or permanent impairment of function (intracranial hemorrhage or embolism), and possibly death. Despite their importance, as well as the known benefit of warfarin, appropriate medication use remains a challenge for patients. Indeed, for vitamin K antagonists, the rates of non-adherence range from 22–58 %. This rate is significant because 34 to 43 % of patients taking warfarin are not in therapeutic range [1], which results in poor clinical outcomes [2]. The history of non-adherence is likely underrepresented in trial outcomes research. As a real world consequence, patients receiving chronic warfarin therapy who have poor anticoagulation control are at increased risk for adverse events [3]. In large studies, the rate of premature discontinuation of new direct oral anticoagulants (DOACs) for no apparent reason is reported to be between 3 and 14.3 % [1]. Contrary to warfarin, one important benefit of DOACs is that they do not require routine coagulation control due to their predictable pharmacokinetic and pharmacodynamic profiles [4]. However, there is agreement that some clinical circumstances require measuring the anticoagulant effect of the DOAC (5) (*e.g.*, preparation for surgery; major bleeds, compliance and/or effect checks, renal impairment). However, concentrations of DOACs and their effect on coagulation are dependent on the pharmacokinetics of the drug and on the concomitant presence of potent P-glycoprotein inhibitors or agonists. To improve efficacy and safety, dose adjustment based on the therapeutic effect may be more appropriate than fixed-dose therapy. The anti-Factor Xa method using chromogenic assay and expressed as μg/L measures the drug concentration and not the intensity of the drug’s anticoagulant activity, and a higher or lower than expected DOACs plasma level does not necessarily indicate an increased risk of bleeding or thrombotic complications [4, 5].

Then it is imperative in daily practice to periodically evaluate dosage to ensure safety and effectiveness. Coagulometric tests based on Russell’s viper venom has been proposed as potential methods to evaluate DOACs as they are sensitive to both classes of these inhibitors [6–8].

## Methods

### Normal donors

Twenty healthy volunteers (12 women and 8 men) with no history of thromboembolic or hemorrhagic diseases, cardiac, renal, hepatic, or malignant diseases, were required to be drug free for 10 days before the study. Only platelet poor plasma from subjects with a normal prothrombin time, activated partial thromboplastin time and thrombin time that fulfilled the inclusion criteria were used.

### Patients

This study included patients with no history of hemorrhagic diseases receiving prolonged oral therapy with rivaroxaban. The review board of the Centro de Trombosis de Buenos Aires approved the study. A total of 158 at peak and 158 at trough measurements of DVVconfirm were performed in 58 patients (36 men, 22 women; mean age, 66.8 ± 14.8 years; median age, 69 years). Patients were referred to our clinic after their own physician recommended drug use due to deep venous thrombosis (26 patients, 3 with pulmonary embolism), portal vein thrombosis (1 patient), mesenteric vein thrombosis (1 patient), atrial fibrillation (30 patients 1 patient with coronary stenting). Fifty patients were treated with 20 mg rivaroxaban per day, and 8 with 15 mg/day. Medication was taken at the same hour each morning. Patients were required to have received therapy for ≥14 days before entering the study. Study complies with the Declaration of Helsinki and informed consent was obtained from all patients. Among the 58 patients Rivaroxaban plasma concentration (using chromogenic assay and expressed as μg/L) at peak and at trough were performed in 30 patients (10 men, 20 women) and compared with DVVconfirm assay (data were expressed as ratio of patient plasma/normal plasma values) (R-C).

### Hemostasis tests

Venous blood was drawn from the antecubital vein and mixed with 0.11 mol/L sodium citrate (1:10 v/v). Blood samples for assays were obtained at the peak of drug activity (mean, 2.05 ± 0.23 h; median, 2.1 h) and during the activity trough 0–2 h before the next dose (mean, 23.6 ± 1.19 h; median, 23.7 h). Tests were performed within 3 h of sampling. Platelet-poor plasma (PPP) was obtained by centrifuging blood samples at 900 × g for 15 min. DVVconfirm® was used to measure citrated PPP using the reagents from Sekisui Diagnostics (Stanford, USA) following the manufacturer’s indications using a coagulometer ST4 (Diagnostica Stago, Asnieres, France). DVV-confirm assay results are reported in seconds of time to clot and the seconds are used to determine the ratio expressed as R-C values.

Rivaroxaban-containing calibrators (241 μg/L of rivaroxaban) were reconstituted with 1 ml pooled normal plasma to obtain a concentration of 241 μg/L, which was further diluted serially with the same pooled normal plasma to obtain concentrations between 0 and 241 μg/L. For the *in vitro* spiking studies rivaroxaban-containing calibrators (purchased from Stago (Diagnostica Stago, Asnieres, France) were used for both the DVV-confirm and the chromogenic assays.

Rivaroxaban chromogenic assays were performed according to Samama *et al.* [9] using STA Liquid anti-FXa assay controls and calibrators from Stago (Diagnostica Stago, Asnieres France) with a STA analyzer.

### Statistical analysis

Qualitative data are expressed as percentages, whereas quantitative data are shown as mean ± SD. The differences between “peak” vs. “trough” values were explored using a paired Student’s *t*-test. The correlation between rivaroxaban concentration and R-C was determined using several different models, including the linear Pearson approach as well as other, more complex, models (exponential, quadratic, cubic, etc.). The corresponding R-square coefficients were obtained. The simplest model providing the most significant R-square coefficient (compared with the linear Pearson model) was considered to have the best fit. All statistical analyses were performed using SPSS, and p-values below 0.05 were considered significant (two-tailed).

## Results

### *In vitro* spiking studies

**Fig. 1:**
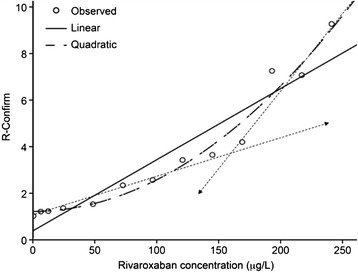
*In vitro* spiking studies. Correlation between rivaroxaban concentration and R-C. For *In vitro* spiked studies known of rivaroxaban concentrations between 0 and 241 μg/L were used. Statistically significant R-Squares of 0.910 (*p* < 0.0001) using a linear correlation and an R-Square of 0.971 using a quadratic equation (*p* < 0.0001) indicate an excellent correlation between rivaroxaban concentration and R-C test. Apparently the curved line has two lineal components (dashed lines)

For *In vitro* spiked studies known concentration of rivaroxaban was used. The correlation between rivaroxaban concentration and R-C using a simple linear correlation yielded an R-square coefficient of 0.910 (*p* < 0.0001) (Fig. 1); using a quadratic model, the R-square coefficient was 0.971 (*p* < 0.0001) (Fig. 1). The corresponding parameters and constant are depicted in Table 1. As mentioned above, the “goodness of fit” was significant using the quadratic approach. Overall, these correlation coefficients indicate an excellent correlation between rivaroxaban concentration and R-C test. As seen in Fig. 1, it seems feasible to interpret the curvilinear quadratic graph as being composed of two linear components (dashed lines). This finding warranted further exploration.

**Table 1:** Model summary and parameter estimates

Equation	Model summary					Parameter estimates		
	R square	F	df1	df2	Sig.	Constant	b1	b2
Linear	.901	100.405	1	11	.000	.371	.031	
Quadratic	.971	169.965	2	10	.000	1.195	.000	.000

Dependent variable: R-C

The independent variable is Rivaroxaban concentration

#### Measurements in plasma patients receiving oral therapy with rivaroxaban

The correlation between rivaroxaban concentration measured as aFXa in patients plasma and R-C is shown in Table 2. The correlation between plasma rivaroxaban concentration and R-C at trough (24 h of drug ingestion) yielded a R coefficient of 0.688 (R2: 0.473, *p* < 0.001) at the linear model; at peak (after 2 h of drug ingestion) the strength of the association was weaker, with an R linear correlation coefficient of 0.39 (R2: 0.15; *p* = 0.03).

**Table 2:** Chromogenic substrate test

Correlation at trough (24 h)	R-C
aFXa Rivaroxaban	0,6881
	*P* = 0.0001
Correlation at peak (2 h)	
aFXa Rivaroxaban	0,3908
	*P* = 0.033

Coefficient of correlations between R-C and rivaroxaban in plasma

R-C Ratio patient plasma / normal plasma

#### Rivaroxaban-test coagulometric method

**Fig. 2:**
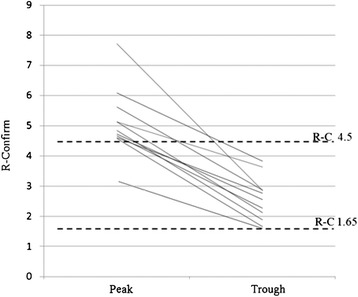
DOAC-test coagulometric method. Ten plasmas among 158 assayed for R-C value were above R-C of 4.5 proposed as cut-off for increased bleeding tendency. Patients were above range at peak time only. The value at peak R-C is 5.39 ± 1.01, with a median of 5.13. Peak vs. trough values: *p* < 0.001

**Fig. 3:**
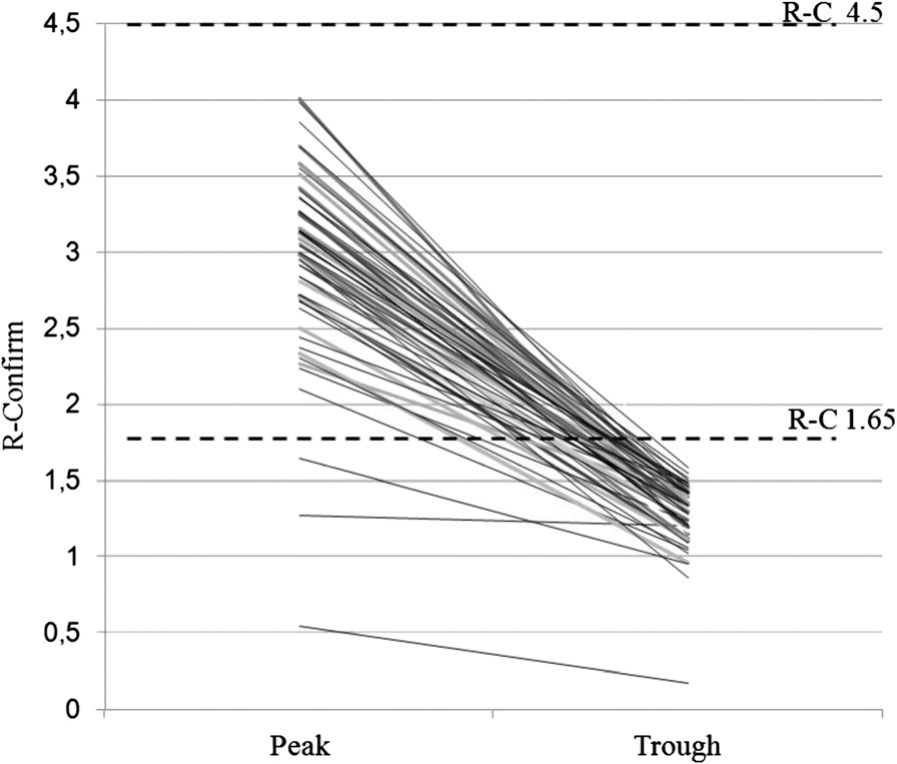
Sixty six plasma samples among 158 assayed for R-C value were below an R-C of 1.65, which was proposed as the cut-off for increased pro-thrombotic tendency. Three patients were below range at both peak and trough. The value at trough R-C is 1.30 ± 0.18, with a median of 1.35. Peak vs. trough values: *p* < 0.001

To evaluate the rivaroxaban coagulometric method, where the patient should be at both peak and trough times the Russel’s viper venom-confirm® method was used in 158 plasma samples from 58 patients. Considering cut-offs of 1.65 and 4.5 as limits of therapeutic levels, 10 determinations (6.3 %) (Fig. 2) were above the bleeding risk level (R-C 5.39 ± 1.01, median 5.13) and 66 tests (42 %) (Fig. 3) were below the theoretical prevention cut-off (R-C 1.31 ± 0.18, median 1.35).

## Discussion

Although quantitative anti-FXa chromogenic assay has been proposed to measure rivaroxaban plasma levels for patients under rivaroxaban therapy, it is not widely performed or available [4–6]. With the absence of widely accepted therapeutic target levels, speculative cut-off levels for safety and prevention were used in the current manuscript.

Taking into account the hemorrhagic level of patients with Factor X deficiency as well as their haemostatic level, this prospective study based on an empirically validated numerical model was designed to investigate cut-off levels in patients receiving rivaroxaban therapy.

Rivaroxaban blocks FX activity, and the unbounded FXa remains available in the clotting system. Even if clinical trials prove the safety of the fixed dose regimen, patients should get frequent follow-up attention by nurses regarding any side effects that do not occur in daily practice.

The cut-off for prevention was based on the plasma activity of vitamin K-dependent clotting factors measured in patients on warfarin, a treatment with a target INR range of 2.5 ± 0.3. In Dargaud *et al.* study [10] the Factor X level in patients without complications was 19 ± 10 IU dL^−1^, and the level in patients with thrombosis was 16 ± 5 IU dL^−1^. Moreover, bleeding is infrequent in patients with F X levels above 20 % [11].

Congenital or acquired FX deficit allows us to speculate on the safety of patients undergoing rivaroxaban therapy. Bleeding symptoms tended to correlate with FX levels: mild (>6–10 %), moderate (1–5 %) or severe (<1 %). Severe clinical symptoms, such as intracranial hemorrhage, gastrointestinal bleeding and hemarthrosis, are not common in patients with FX levels >2 % [12]. Patients with FX levels <10 % present with mucocutaneous bleeding, whereas those with moderate to severe deficiency may have symptoms such as hemarthrosis, intracranial hemorrhage, and gastrointestinal bleeding. These patients experience spontaneous bleeding when their plasma Factor X concentration is below 1 % [13, 14]; patients with higher levels of Factor X can also bleed in a traumatic situation. Patients with amyloidosis [15] developed severe gastrointestinal bleeding as a result of a significant decrease in Factor X levels, but a patient who presented with spontaneous retroperitoneal bleeding had 22 % FX activity [16].

Based on these data and that reported in our previous publication [7], Factor X concentration curves were created. Based on these curves, 1.56 % FX corresponded with a R-C of 4.5, and 20 % FX corresponded with a R-C of 1.65. These values were designated as cut-offs for safety and thromboembolic prevention in the current study.

An optimal drug level should be obtained to achieve the correct balance between antithrombotic efficacy and bleeding risk. Brummel-Ziedins *et al.* [17] found a marked interindividual variation of FXa generation in a healthy population (from 49 to 163 %) and in individuals with a known DVT history (from 58 to 174 %). with statistically significant differences between groups. These differences were also related to gender, BMI and oral contraceptives [17]. Patients taking 20 mg rivaroxaban had a daily concentration peak that varied between 177 and 361 μg/L and a trough of 9.02 to 147 μg/L [18, 19]. Similarly, at a dose of 10 mg, the daily concentration peak was 75.1 to 177 μg/L and a trough of 1.35 to 37.2 μg/L. These interindividual variations were also observed with other DOACs [20, 21].

In the current study based on *in vitro* spiking studies, it is apparent that the DOAC-rivaroxaban coagulometric method is suitable for measuring non-rivaroxaban bounded plasma-activated Factor X *ex vivo*. The results obtained with spiked plasma may not be similar to those obtained from *ex vivo* patient plasma due to the absence of drug metabolites. At high concentrations, rivaroxaban or its metabolites may have additional effects on clotting mechanisms that are currently unknown [22].

Thus, the question arises over whether rivaroxaban concentrations measured by an anti-FXa chromogenic assay indicate the extent of clotting inhibition mechanisms as well as a coagulometric assay. Given the marked variation between healthy individuals with thrombosis as well as patients with liver, kidney or bowel disease, one must assume that a significant fraction of patients will be exposed to either very low or very high drug levels [23]. Safety in daily practice could be improved with an easy-to-use coagulometric method. In the absence of widely accepted therapeutic target levels, we speculate cut-off points for identifying patients with an increased risk of hemorrhage, R-C ≥4.5, or those exhibiting a low anticoagulation effect, R-C of ≤1.65, as potential cut-offs of a pro-thrombotic state. Using the proposed therapeutic range for 158 plasma samples, 10 (6.5 %) were above the level of bleeding tendency at the peak (R-C 5.39 ± 1.01, median 5.13) and 66 (42 %) were below the prevention cut-off at the trough (R-C 1.31 ± 0.18, median 1.35), which indicates that treatment with rivaroxaban warrants dose adjustment. The paper by Beyer-Westendorf *et al.* [24], describe that in real life, rates of rivaroxaban-related major bleeding may be lower and that the outcome may at least not be worse than that of major vitamin K antagonist bleeding. We assume that in patients on rivaroxaban, routine measure of anticoagulant activity using a coagulometric method as R-C could lower risk of bleeding and/or thromboembolism compared with no routine coagulation monitoring. Our proposal should be considered hypothesis-generating; it requires further clinical studies to show that in real life, this monitoring strategy actually reduces bleeding and risk of ischemic stroke.
